# Atraumatic Spontaneous Splenic Rupture With Unknown Etiology

**DOI:** 10.7759/cureus.45364

**Published:** 2023-09-16

**Authors:** Humberto Perez, Han Sol Jeong, Jason Smith DO

**Affiliations:** 1 General Surgery, Lincoln Memorial University DeBusk College of Osteopathic Medicine, Miami, USA; 2 General Surgery, Lincoln Memorial University DeBusk College of Osteopathic Medicine, Centreville, USA; 3 General Surgery, AdventHealth Florida, Mt. Dora, USA

**Keywords:** emergency department visits, traumatic splenic rupture, general surgery consult, total splenectomy, atraumatic splenic rupture

## Abstract

Splenic rupture of all causes is a potentially life-threatening event for patients. The infrequency of atraumatic splenic rupture (ASR) poses a significant diagnostic challenge due to atypical findings. ASR is commonly due to a spleen with an underlying disease process such as malignancy, infection, coagulopathies, or neoplasms. However, ASR without an identifiable cause is rare and poses further complexity. In this case, a 57-year-old woman with a history of hypertension presented to the emergency department complaining of chest pain and was found to have a splenic hematoma. She underwent splenic artery embolization due to her continued hemodynamic instability. The patient was ultimately treated with a splenectomy, as embolization was unsuccessful. Gross pathology revealed no underlying disease processes, nodules, or masses. Splenic hemorrhage due to atraumatic rupture of the spleen is rare and without known pathology. The case illustrates the need for providers to have high clinical suspicion of such a diagnosis to stabilize and surgically manage these patients. Few instances of ASR without an identifiable cause are found in medical literature, and further knowledge of the subject is needed.

## Introduction

Splenic rupture is a potentially life-threatening condition commonly due to blunt abdominal trauma that is extensively covered in the medical literature [1-3]. However, splenic rupture can occur less frequently without apparent trauma, referred to as atraumatic splenic rupture (ASR). ASR is well documented when it is due to an underlying diseased spleen, coagulopathy, infection, or neoplasm [4]. The presentation of ASR has an approximate mortality rate of 12% [5]. ASR lacking the above risk factors or without a known etiology is rare and poorly documented [2,3].

Cases of ASR without the above etiologies are related because they lack the typical clinical presentation expected among these patients and present a challenge in determining appropriate management. Splenic rupture is a clinical diagnosis confirmed by a CT scan or laparotomy in hemodynamically unstable patients. There are well-established CT or ultrasound grading systems that can help guide the appropriate management of patients with ASR.

## Case presentation

**Figure 1:**
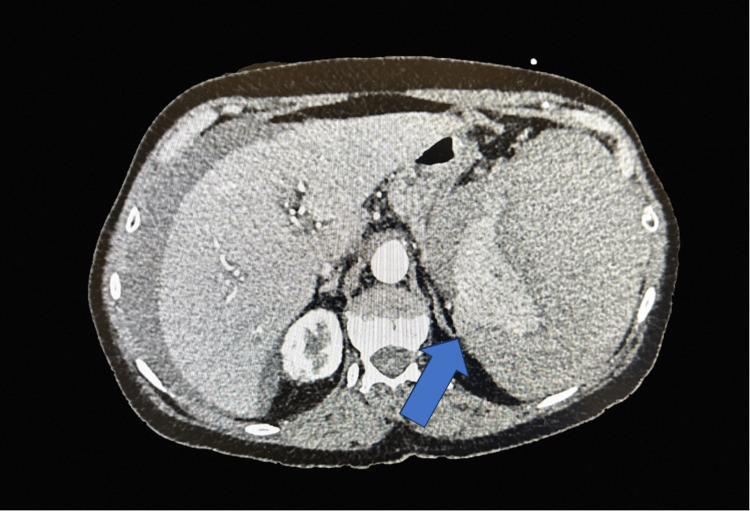
Computed tomography whole abdomen Arrow: Cross-sectional view of the abdomen with findings concerning of splenic hemorrhage with large perisplenic hematoma compressing the splenic parenchyma. Multiple hypodense lesions within the spleen are incompletely characterized.

A 57-year-old female presented to the emergency room complaining of chest pain. Her medical history was notable for hypertension treated with 5 mg of amlodipine once daily and 50 mg of metoprolol tartrate twice daily. Her family history was unremarkable. Her social history was remarkable for her smoking and alcohol use. Equal in timing, she reported chest pain localized to the left lateral side, radiating to the abdomen and left flank. The patient appeared to be in acute distress and diaphoretic. The patient denied any recent trauma. On physical exam, she was tachycardic and had diffuse abdominal tenderness to light and deep palpation. Her abdomen was non-distended, dull to percussion, and had no rebound tenderness. Pertinent laboratory findings included the following: normal hemoglobin (12.2 g/dL), normal blood urea nitrogen/creatinine ratio (15/1.4), and a mildly elevated troponin (19 ng/L, reference range <14 ng/L). A computer tomography angiography (CTA) of the chest, abdomen, and pelvis demonstrated a large peri-splenic hematoma measuring 12.5 cm × 15.4 cm × 13.1 cm with no definite active extravasation (Figure 1).

**Figure 2:**
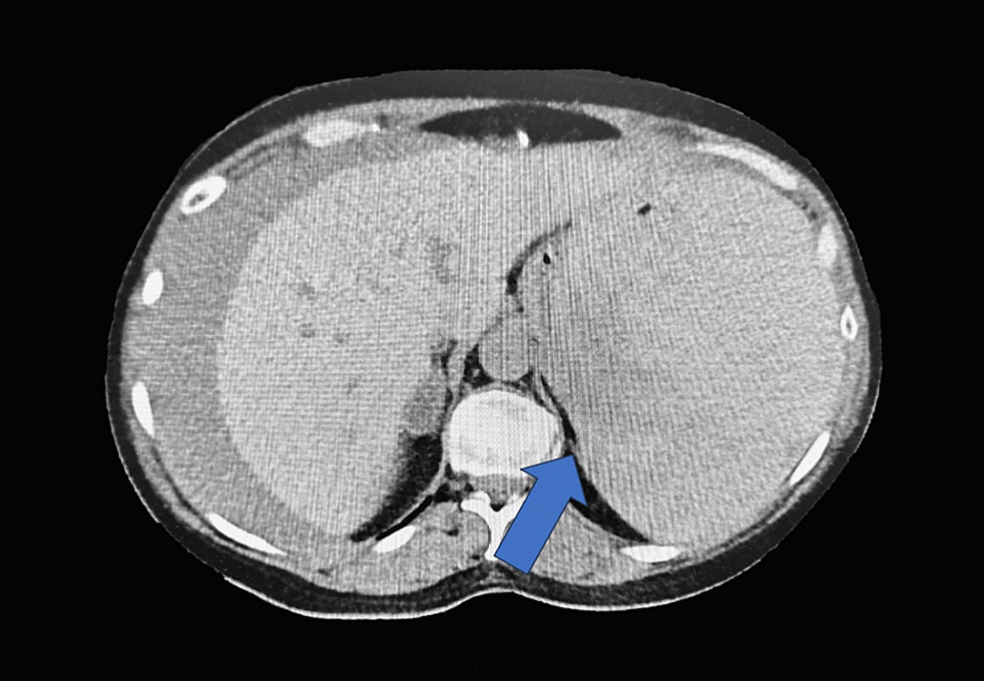
Computed tomography whole abdomen Arrow: cross-sectional view of the abdomen. Spleen not visualized secondary to large perisplenic hematoma. The sentinel hematoma in the left upper quadrant measures 13 cm × 17 cm.

Eight hours later, the patient displayed signs of hemorrhagic shock, including an elevated heart rate and low blood pressure. Repeat hemoglobin decreased to 9.6 g/dL, and a massive transfusion protocol was initiated. The patient underwent a splenic artery embolization; however, she continued to have multiple hypotensive episodes throughout the night. A repeat CTA revealed that the peri-splenic hematoma had increased to 13 cm × 17 cm with a significant mass effect on the stomach (Figure 2).

At that time, the patient underwent an emergency splenectomy, during which approximately 3.5 L of blood was evacuated. The spleen was actively bleeding through multiple fractures. Two units of PRBC and two units of FFP were administered intraoperatively. Gross spleen pathology revealed benign splenic rupture with evidence of hemorrhage, without nodules or discrete masses. There were no splenic artery abnormalities. Postoperatively, the patient was transferred to the ICU, where she remained hemodynamically stable with a positive hemoglobin trend. The remainder of this patient’s hospital course was unremarkable. She was given prophylactic vaccinations, including meningococcal A, meningococcal B, *Haemophilus influenzae* (type b), and pneumococcal 23, and discharged on postoperative day 7.

## Discussion

The majority of splenic rupture cases, overall, are due to blunt abdominal trauma [6]. ASR is a rare phenomenon most commonly due to infectious disease, medical procedures, or hematological causes [7].

Investigation of cases defined as "true" ASR often involves other underlying pre-existing diseases such as diabetes, heart disease, splenic abscess, or malignancy [8]. In this case, the patient's splenic pathology was benign, and there were no known malignancies, recent abdominal surgery, chronic conditions, or anticoagulant medications.

However, the patient's history of hypertension and alcohol use disorder may have contributed to the atraumatic splenic rupture, but there has been no evidence in the literature. To the best of our knowledge, idiopathic atraumatic splenic rupture is a rare occurrence with an incidence of 0.1-0.5% [9].

Treatment considerations for ASR should not take a different approach than those for typical causes of traumatic rupture. Hemodynamic stability is the primary consideration; however, total splenectomy should be considered as a first-line approach in cases with underlying malignancy or where the splenic function is compromised. A more conservative approach involves splenic artery embolization with IR, which was a successful treatment method for a spontaneous splenic rupture in a patient with COVID-19 [10]. Additionally, patients over the age of 40 possess a greater risk of ASR mortality [5], such as with this patient, where initial conservative treatment for embolization failed and the patient ultimately underwent a total splenectomy.

Although ASR is not a common cause of splenic hemorrhage, it should be considered a potential cause, and a total splenectomy should be anticipated.

## Conclusions

Splenic rupture without traumatic or pathological causes is a rare occurrence that can lead to hemorrhagic shock and death. Indeed, spontaneous ruptures are a rare occurrence. Patients with pre-existing conditions such as diabetes or heart failure over 40 presenting signs of diffuse abdominal pain, hypotension, and tachycardia should be considered for splenic rupture. After hemodynamic stability, additional workup for coagulopathies and underlying malignancies or conditions such as amyloidosis should be investigated. Splenic rupture should be considered in the setting of acute abdominal pain, despite no known traumatic or pathologic causes.
